# Octyl-2-cyanoacrylate tissue adhesive without subcuticular suture for wound closure after total hip arthroplasty: a prospective observational study on thirty-two cases with controls for 3 months follow-up

**DOI:** 10.1186/s13018-020-01997-9

**Published:** 2020-10-10

**Authors:** Li-Shen Wang, Xin-Yu Wang, Hao-tian Tu, Yi-Fan Huang, Xin Qi, Yu-Hang Gao

**Affiliations:** Department of Orthopedic Surgery, The First Hospital of Jilin University, Changchun, Jilin, 130021 China

## Abstract

**Background:**

Whether using tissue adhesive alone after subcutaneous suture can close the skin incision with safety as well as cosmetic appearance after total hip arthroplasty was not clear.

**Methods:**

A prospective study was conducted. The same surgical methods were consistent throughout the entire study. After implanting prosthesis, the joint capsule was reconstructed. Fascial and subcutaneous layer were respectively closed by continuous running barbed suture. Patients were randomized allocated to group A with octyl-2-cyanoacrylate tissue adhesive alone, to group B with tissue adhesive after continuous subcuticular suture, or to group C with skin staples. Time of closure, drainage, pain, wound complications, and cosmesis were compared. All data were analyzed statistically.

**Results:**

There was no significant difference in drainage, Visual Analog Scale score or early wound complications between the three groups. However, there was significant difference in time of closure (*P* = 0.013). In pairwise comparison, time of closure in groups A and B was significantly longer than those in group C (*P* = 0.001 and *P* = 0.023, respectively); time of closure in group A was significantly shorter than those in group B (*P* = 0.003). Patient and Observer Scar Assessment Scale total scores were not significantly different at 6 weeks and 3 months postoperatively (*P* = 0.078 and *P* = 0.284, respectively).

**Conclusion:**

Tissue adhesive without subcuticular suture was similar with a combination of subcuticular suture and tissue adhesive as well skin staples in terms of safety and cosmetic appearance after total hip arthroplasty.

## Introduction

A safe technique of wound closure permits rapid incision healing without complication; therefore, it is very important for rapid rehabilitation and quicker discharge of patients from hospital following total hip arthroplasty (THA) [1]. Poorly postoperative incision healing will bring negative effects to patients, including delayed wound healing, prolonged hospitalization, scar hyperplasia, decreased patient satisfaction, affected patient activity and functional recovery, and increased medical costs [2, 3]. In addition, infection of the surgical incision is one of the risk factors for periprosthetic infection which is the most severe complication after THA [4].

Skin staples were the most commonly employed technique of wound closure in in arthroplasty surgery [5]. However, there is evidence indicating that a significantly higher risk of developing a wound infection in patients who undergo hip surgery when the wound is closed with staples rather than sutures [6]. In recent years, octyl-2-cyanoacrylate tissue adhesive has been widely used in the treatment of pediatric skin injury [7]. Tissue adhesive had some potential advantages such as ease of use, painless application, rapid closure, cosmesis, and avoidance of needlestick injuries and removal of the suture or staples [8]. It was also applied in a high volume arthroplasty unit for skin closure and did not increase the risk of surgical site infection in THA [9]. This technique also provided an immediate water tight seal in a sterile operative environment and a barrier to microorganisms; it had good tensile strength, esthetic value, and patient satisfaction [10]. Several studies have compared the efficacy of tissue adhesive, subcuticular suture and skin staples, the results indicated that skin staples had a higher rate of discharge on post-operative days 1 and 3 as well as shorter time of closure, but little difference in the occurrence of complications, scar outcome, or patient satisfaction between the three techniques [11–13].

However, in the mentioned above studies, subcuticular layer were sutured routinely before tissue adhesive was used after THA. A randomized controlled trial comparing the safety of tissue adhesive and subcuticular suture at skin closure after subcutaneous suture of cesarean delivery shown that the two methods had similar results [14]. These results may indicate that tissue adhesive was able to close the skin incision safely independent of subcuticular suture. However, to the best of our knowledge, whether using tissue adhesive alone after subcutaneous suture can close the wound with safety as well as cosmetic appearance after THA was not clear. Therefore, this prospective study was designed to compare these outcomes between techniques of tissue adhesive alone, a combination of subcuticular suture and tissue adhesive and skin staples at wound closure after subcutaneous suture in THA.

## Patients and methods

### Participant recruitment

This prospective study was conducted at our institution between December 2018 and June 2019 in accordance with the Declaration of Helsinki. The Institutional Review Board approved the study (IRB number IRB00008484). The statistical power of this study was based on published literature assuming that the true difference between the means was at least one standard deviation of the variable score, a sample size of 66 (22 per group) would reveal differences at the 5% level of significance, and with a power of 0.9. Therefore, three groups of 30 subjects would allow for a 20% dropout during follow-up [13]. Recruitment occurred during preoperative visits with one attending orthopedic residents (WLS) involved in the study. Patients meeting inclusion criteria were asked if they would like to be involved in the study and those who were interested signed an informed consent form. Inclusion criteria included being scheduled to undergo a primary unilateral THA due to osteonecrosis of the femoral head or hip osteoarthritis. Exclusion criteria included patients with a previous incision or infection in the operative field, a history of keloid formation, diabetes, hypoproteinemia, regular anticoagulation therapy, lower extremity vascular disease, or skin allergy.

### Surgical and suturing methods

The surgical methods were consistent throughout the entire study. All procedures were performed by one surgeon (QX) through a posterolateral approach. Cementless stem (Depuy, Corail® Total Hip System) was used in all hips. After implanting prosthesis, the joint capsule was reconstructed. No drain was used in the deep layers in all wounds. Fascial (antibacterial polydioxanone, 0 symmetric PDS PlUS; Ethicon Inc.) and subcutaneous layers (violet monofilament synthetic absorbable polydioxanone, 2-0 Spiral PDO; Angiotech Puerto Rico, Inc.) were respectively closed by continuous running barbed suture. The resident (WLS) had used barbed suture on twenty cases prior to the study to become comfortable using it. To be noted, suture technique described as previous literature [15] that upon reaching the end of the wound a few redundant throws were inserted back toward the center of the wound to secure the suture in place was very important for patients with strong body status, since dehiscence of barbed suture on fascia occurred in two cases with strong body status at the end of suture when we did not make more throws back toward. After following this tip of suture, no such compilation occurred in the left cases until this study began. Allocation of wound closure took place in the operating room after subcutaneous suture by the random drawing of numbers by a personnel not involved in this study. Patients were allocated to group A with octyl-2-cyanoacrylate tissue adhesive (Johnson and Johnson, New Brunswick, New Jersey) alone in accordance with the manufacturer’s instructions (one entire pen-applicator’s worth of Dermabond applied to the wound followed by air drying for 30 s, placement of a second layer with a second entire pen applicator, and a second air-drying period of 60 s), to group B with tissue adhesive after continuous 3.0 subcuticular absorbable poliglecaprone suture (Monocryl, Johnson and Johnson), or to group C with skin staples (Proximate®, Ethicon Endo-Surgery, Cincinnati, OH).

### Data collection

Time of closure was recorded from the first insert of subcutaneous suture to the completion of wound closure. These data were recorded by a medical student (HYF) in the operating room during closure. A standardized dressing consisting of five gauzes stacked directly on top of one another was applied for all patients. Closure and dressing application for all enrolled patients were performed by the resident (WLS). Length of the incision, leg length discrepancy, and limb length changes were also recorded. All patients were treated with the same postoperative protocol: The patients were allowed to stand up at the sixth hour after operation, and began to walk with crutches from the first day postoperatively. Oral rivaroxaban for anticoagulation and analgesic treatment were scheduled routinely.

The incision were evaluated on the first, second, third, fifth, seventh, and fourteenth days after operation. In groups A and B, the incision were evaluated after uncovering the tissue adhesive and did not change the dressing, but cleaned with wet tower when needed, then the tissue adhesive was covered as before; in group C, the dressing was changed every 3 days. The uncovered gauzes were collected in each group until there was no exudate. The novel technique to calculate the drainage was followed as previous literature described: Analysis was performed by staff (GYH) blinded to group allocation to quantify the exudate on the gauze dressings. Each individual layer of gauze was examined by illuminating the gauze through a glass panel, placing a standardized graph paper over it, and recording the number of boxes of graph paper overlapping any drainage stain on the gauze. The graph paper boxes measured 0.1 square inches each and exudate in any portion of a box was counted as a complete box. The number of exudate boxes recorded for each layer of gauze was combined to quantify the volume of drainage left in the 5 layers of gauze from each wound [16].

Visual Analog Scale (VAS) scores at the third day postoperatively and early wound complications (within 1 week after operation) including fat liquefaction, stitch exclusion, split incision, allergic reaction, and infection were recorded.

Skin scars were evaluated by the Patient and Observer Scar Assessment Scale (POSAS) total score at 6 weeks and 3 months after operation. This score included five observer scar assessment scale and six patient scar assessment scale. Two observers (WXY and THT) attended and evaluated separately. The mean value of the two scores was recorded as the final score for skin scar. Pain, itch, color, stiff, thickness, and irregularity were assessed by patients themselves [17].

### Statistical analysis

All data between the three groups were analyzed statistically. The Kolmogorov–Smirnov test was used to evaluate the normality of the groups’ data distribution. The Kruskal-Wallis test was used to compare the continuous variables across three groups. Pearson chi-squared test compared the observed frequencies of the categorical data, otherwise Fisher’s exact test was used. Mann-Whitney test was used to make pairwise comparisons between groups where an overall significant difference was found. The two-tailed values of *P* < 0.05 were considered statistically significant. All statistical analyses were performed by using SPSS Statistics 22.0 (SPSS Inc., Chicago, Illinois).

## Results

**Fig. 1:**
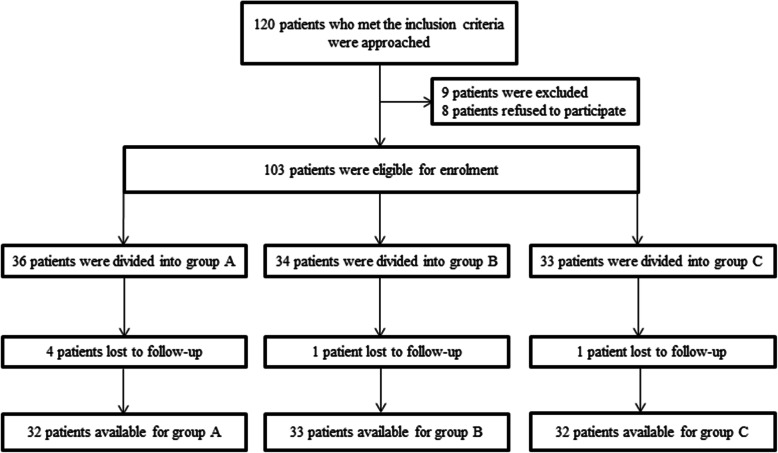
The CONSORT flow diagram

During the recruitment period, a total number of 120 patients who met the inclusion criteria were approached, of whom nine patients were excluded and eight patients refused to participate in this study, left 103 cases were eligible for enrolment. Thirty-six, 34, and 33 cases were divided into groups A, B, and C, respectively. Four cases in group A, one case in group B, and one case in group C were lost to follow-up for personal reasons. Therefore, 32 patients in group A, 33 patients in group B, and 32 patients in group C were available for this study (Fig. 1). The patients’ details were presented in Table 1. There was no statistically significant difference between the three groups in terms of gender, age, BMI, hemoglobin, platelets, length of the incision, leg length discrepancy, or limb length changes.

**Table 1:** Patient characteristics

	Group A (*n* = 32)	Group B (*n* = 33)	Group C (*n* = 32)	*P* value
Gender (male/female)	24/32 (75)	19/33 (57.6)	18/32 (56.3)	0.222^b^
Age (years)	54.6 (32 to 73)	55.4 (29 to 72)	52.7 (29 to 74)	0.795^a^
BMI (kg/m^2^)	25.7 (19 to 36)	24.5 (20 to 29)	24.7 (18 to 28)	0.659^a^
Hemoglobin (g/L)	150.8 (121 to 192)	141.5 (119 to 162)	142.1 (94 to 186)	0.064^a^
Platelets (10^9^/L)	243.2 (100 to 515)	247.1 (178 to 419)	256.5 (146 to 480)	0.590^a^
Length of the incision (cm)	11 (9.5 to 12)	10.8 (9.7 to 13)	11.4 (10 to 12.8)	0.840^a^
Leg length discrepancy (cm)	0.2 (0 to 0.6)	0.2 (0.1 to 0.5)	0.3 (0.1 to 0.6)	0.753^a^
Limb length change (cm)	0.8 (0.5 to 1)	0.6 (0.2 to 0.8)	0.9 (0.3 to 1.1)	0.836^a^

Numerable data were presented as median (range)

^a^Kruskal-Wallis test

^b^Pearson chi-squared test

Comparisons of time of closure, drainage, VAS score, wound complications, and POSAS scores were presented in Table 2. There was no significant difference in drainage, VAS score at third day postoperatively or wound complications between the three groups. However, there was a significant difference in the time of closure (*P* = 0.013). In pairwise comparison, time of closure in groups A and B were significantly longer than those in group C (*P* = 0.001 and *P* = 0.023, respectively); time of closure in group A was significantly shorter than those in group B (*P* = 0.003). POSAS total scores were not significantly different between the three groups at 6 weeks or 3 months postoperatively (*P* = 0.078 and *P* = 0.284, respectively). However, in patient score section at 6 weeks, there was a significantly difference between the three groups (*P* = 0.030). In pairwise comparison, patient scores were significantly lower in group A than that in group C (*P* = 0.028).

**Table 2:** Comparisons of time of closure, drainage, pain, wound complications, and cosmesis

	Group A (*n* = 32)	Group B (*n* = 33)	Group C (*n* = 32)	*P* value	*P* value_A vs B_	*P* value_B vs C_	*P* value_A vs C_
Time of closure (min)	9.1 (7 to 11 )	13 (10 to 15)	7 (6 to 9)	0.013^a^	0.003^b^	0.023^b^	0.001^b^
Drainage (number of boxes)	210.5 (15 to 948)	209.9 (41 to 450)	201.3 (35 to 593)	0.530^a^			
VAS score at 3rd day postoperatively	2.1 (0 to 4)	1.9 (1 to 4)	2.4 (1 to 4)	0.144^a^			
Fat liquefaction	2	3	5	0.502^c^			
Stitch exclusion	4	3	2	0.756^c^			
Split incision	0	0	0				
Allergic reaction	0	0	0				
Infection	0	0	0				
POSAS total score at 6 weeks postoperatively	24.7 (22 to 33)	24.8 (21 to 29)	25.7 (22 to 32)	0.078^a^			
Patient score	12.5 (10 to 17)	12.6 (10 to 15)	13.1 (11 to 16)	0.030^a^	1^b^	0.230^b^	0.028^b^
Observe score	12.3 (10 to 16)	12.2 (9 to 14)	12.3 (10 to 17)	0.301^a^			
POSAS total score at 3 months postoperatively	24.4 (20 to 30)	24.2 (20 to 28)	25.0 (19 to 32)	0.284^a^			
Patient score	12.3 (10 to 16)	11.9 (10 to 14)	12.6 (10 to 16)	0.067^a^			
Observe score	12.2 (10 to 14)	12.3 (9 to 16)	12.4 (9 to 16)	0.718^a^			

Numerable data were presented as median (range)

^a^Kruskal-Wallis test

^b^Non-parametric Mann-Whitney *U* test

^c^Fisher’s exact test

## Discussion

The major finding of this study was that using tissue adhesive without subcuticular suture was with safety and cosmetic appearance of scars for wound closure after THA. Khan et al. in their prospective randomized, controlled trial found that with THA there was no significant difference between the groups for either early or late complications, closure of the wound with skin staples was significantly faster than with tissue adhesive or subcuticular suture [13]. However, in this study, the deep tissues were closed in a standard manner using continuous 1 Vicryl for the deep fascia and 2.0-vicryl for the deep dermal layer. Livesey et al. conducted a randomized controlled trial to compare the outcomes of skin adhesive and staples for skin closure in THA [12]. They reported similar results on cosmetic appearance of scars at 3 months, the occurrence of complications, or patient satisfaction. They also used three layers of sutures prior to skin closure method: the fascia lata was closed with continuous no. 2 Vicryl, fat with interrupted no. 1 Vicryl, and the deep dermal layer with 00 Vicryl. Glennie et al. reported that there was no significant difference in POSAS scores at 6 weeks or 3 months, VAS pain scores, length of stay, or total cost, but the staple group had a higher rate of discharge on postoperative days 1 and 3 as well as a 1.6-min shorter time of closure [11]. They also sutured the muscular and fascial layers with no. 1 Vicryl and closed the subcutaneous layer with 2-0 interrupted monocryl suture, after that, applied uninterrupted subcuticular monocryl for the dermal/epidermal layer before tissue adhesive used. In our study, tissue adhesive was used alone for wound closure after subcutaneous suture in a series of patients with THA, and the results of drainage and early wound complications were consistent with previous studies; therefore, we may concluded that tissue adhesive without subcuticular suture was safe for wound closure after THA.

Reliable sutures for fascial and subcutaneous layers were obliged to applying tissue adhesive without subcuticular suture. As we have mentioned above in the method section, dehiscence of barbed suture for fascial layer occurred in two robust cases before this study began. In the two cases, we found that increased drainage was correlated with the amount of exercise after the patients were scheduled to walking postoperatively, and ultrasonic examination indicated that there were subcutaneous effusions. When the incision was reopened, we found that barbed suture on fascia failed in the proximal part of the incision. After we sutured the fascial layer and reversely inserted a few redundant throws back toward the center of the wound, no more complication occurred after that. Barbed sutures were associated with shorter closure times, shorter operative times, and larger cost savings per procedure as well as comparable wound complication rates for deep closure after total joint arthroplasty [18, 19]. However, Campbell et al. used to report a higher rate of dehiscence when using barbed sutures for superficial wound closure in total knee arthroplasty which requires more activities than THA [20]. The barbed suture might cut into the muscular fibers during intensive early exercise in patient with strong body status.

Cosmetic appearance was one of the potential advantages of tissue adhesive for skin closure and affected the patient’s postoperative satisfaction [21]. However, most of the evaluations on scar of incision in THA were comparable between tissue adhesive and skin staples in previous studies. Although the patient scores were significantly different at 6 weeks, POSAS total scores in this study shown similar results at the evaluations of 6 weeks and 3 months postoperatively. In addition, this study firstly demonstrated that there was no difference on scar between using tissue adhesive with and without subcuticular suture. Non-tension suture in skin closure was important for scar formation of the surgical incision [22]. Leg length discrepancy and limb length changes were far from rare after THA, which in many cases will change the tension of the incision and may adversely affect the incision closure. In this study, leg length discrepancy and limb length changes were not significantly different between the three groups. This result may indicate that reliable barbed sutures for fascial and subcutaneous layers were able to effectively reduce the tension of skin closure when tissue adhesive was applied.

There were some limitations in this study. Firstly, the sample size was calculated to find difference on educate according to previous literature. However, due to the low incidence of wound complications [19], no severe complication was found in our patients. Further studies are needed to confirm the rate of complication when tissue adhesive was applied alone for skin closure. Secondly, there are more lost visits in group A than in group B or C. However, we have taken this into account in the design of this study, and recruited more patients than the calculated minimum sample size. The final follow-up rate can reach more than 80%; therefore, these losses of visits may not affect the results. The last but not the least, we did not enroll patients with total knee arthroplasty in this study. Since the tension on incision and requires of motion when these patients do physical exercise were much higher than those with THA, subcuticular suture were obliged to be used before skin closure.

In conclusion, tissue adhesive without subcuticular suture was with safety and skin cosmetic appearance after THA. This study may indicate a more efficient way that can reduce time of closure and cost in wound closure after THA.

## Data Availability

Not applicable

## Abbreviations

THA: Total hip arthroplasty
VAS: Visual Analog Scale
POSAS: Patient and Observer Scar Assessment Scale
